# Post-translational modifications confer amphotericin B resistance in *Candida krusei* isolated from a neutropenic patient

**DOI:** 10.3389/fimmu.2023.1148681

**Published:** 2023-03-01

**Authors:** Li Zhang, Jinzhou Xiao, Mingwei Du, Wenzhi Lei, Weiwei Yang, Xiaochun Xue

**Affiliations:** ^1^Institute of Dermatology, Naval Medical University, Shanghai, China; ^2^Department of Cardiology, Shuguang Hospital Affiliated to Shanghai University of Traditional Chinese Medicine, Shanghai, China; ^3^Shanghai Key Laboratory of Traditional Chinese Clinical Medicine, Shanghai, China; ^4^Department of Thoracic Surgery, Shanghai Pulmonary Hospital, School of Medicine, Tongji University, Shanghai, China; ^5^Shanghai Engineering Research Center of Lung Transplantation, Shanghai, China; ^6^Department of Pharmacy, 905th Hospital of PLA Navy, Shanghai, China

**Keywords:** *Candida krusei*, neutropenia, amphotericin B, resistance, succinylation

## Abstract

Neutropenia is a common complication in the treatment of hematological diseases and the most common predisposing factor for invasion by fungi, such as *Candida krusei*. Recent studies have shown that *C. krusei*, a life-threatening pathogen, has developed resistance to amphotericin B (AMB). However, the mechanisms that led to the rapid emergence of this AMB-resistant phenotype are unclear. In this study, we found the sensitivity for AMB could be promoted by inhibiting histone acyltransferase activity and western blot analysis revealed differences in the succinylation levels of *C. krusei* isolated from immunocompromised patients and of the corresponding AMB-resistant mutant. By comparative succinyl-proteome analysis, we identified a total of 383 differentially expressed succinylated sites in with 344 sites in 134 proteins being upregulated in the AMB-resistant mutant, compared to 39 sites in 23 proteins in the wild-type strain. These differentially succinylated proteins were concentrated in the ribosome and cell wall. The critical pathways associated with these proteins included those involved in glycolysis, gluconeogenesis, the ribosome, and fructose and mannose metabolism. In particular, AMB resistance was found to be associated with enhanced ergosterol synthesis and aberrant amino acid and glucose metabolism. Analysis of whole-cell proteomes, confirmed by parallel reaction monitoring, showed that the key enzyme facilitating lysine acylation was significantly upregulated in the AMB-resistant strain. Our results suggest that lysine succinylation may play an indispensable role in the development of AMB resistance in *C. krusei.* Our study provides mechanistic insights into the development of drug resistance in fungi and can aid in efforts to stifle the emergence of AMB-resistant pathogenic fungi.

## 1 Introduction

Neutropenia, a common complication in the treatment of hematological diseases and malignant tumors, is characterized by a reduction in neutrophil count (to < 1,500/mm^3^) and is a risk factor for candidiasis in immunocompromised patients (1, 2). Although *Candida albicans* is the predominant agent of invasive yeast infections, a marked increase in the incidence of infections by other *Candida* spp. has been recently reported among neutropenic patients (3–6). *Candida krusei*, known as *Pichia kudriavzevii* or *Issatchenkia orientalis*, is an opportunistic pathogen that can cause local or disseminated infections. In immunocompromised patients, especially among those with hematologic malignancies or neutropenia, or those that are solid-organ or hematopoietic stem-cell recipients, *C. krusei* causes life-threatening infections, especially owing to extensive comorbidities in the patient population and its resistance to standard antifungal therapy (7, 8). Mortality rates associated with *C. krusei* fungemia are high: up to 49% with *C. krusei* vs. 28% with *C. albicans* (9–11).

For neutropenic patients, echinocandins, voriconazole, or amphotericin B (AMB) is recommended for the treatment of *C. krusei*-associated candidemia according to the Infectious Diseases Society of America (IDSA). Compared with echinocandins and other drugs, AMB is currently the best performing and least expensive treatment option for patients with neutropenia and has shown significantly improved survival rates in a recent clinical study (12). Unfortunately, *C. krusei* has already developed some resistance to AMB. Several studies from different regions revealed a minimum inhibitory concentration (MIC)-90 of AMB to *C. krusei* of 8 μg/mL, which was eight times greater than that for *C. albicans* (13–16). However, the specific mechanism leading to AMB resistance in *C. krusei* remains unclear, and this retards any efforts towards developing effective clinical treatments.

Recent developments in mass spectrometry (MS)-based proteomics provide a promising approach for identifying the related molecular mechanisms. Proteomics is a sensitive and robust approach for identifying proteins and pathways associated with fungal pathogenesis, drug resistance, modulation of host immune response, and the discovery of drug targets (17–19). For example, comparative proteomics has revealed that energy metabolism and cell stress were related to acquired fluconazole resistance in *C. albicans* (19). Post-translational modifications (PTMs) have also attracted attention because of their vital roles in fungal growth, virulence, stress responses, and drug resistance (20, 21). Increasing evidence indicates that PTMs, especially acetylation, contribute to the development of drug resistance in different fungi (22, 23). However, no study has considered the role of PTMs in the development of drug resistance in *C. krusei*.

In this study, we first established an AMB-resistant *C. krusei* strain (Z748R) through treatment with gradually increasing concentrations of AMB. We found that PTMs and proteome profiles were both associated with the development of AMB resistance. Among the PTMs, succinylation was an essential modification. Our study demonstrated for the first time that the sensitivity of *C. krusei* to AMB might mainly be associated with PTMs, which in turn revealed a potential inducible drug resistance mechanism of *C. krusei*; this could inform responsible drug use in clinical practice to avoid the outbreak of antibiotic-resistant fungi and infection of immunocompromised patients.

## 2 Materials and methods

### 2.1 Strains and culture conditions

We used *C. krusei* strains (Z748, Z571, and Z183) provided by the Shanghai Pulmonary Hospital, which were identified by CHROMagar Candida medium and internal transcribed spacer (ITS) sequencing. We generated the AMB-resistant strain (Z748R) from the Z748 strain through continuous subculturing in Yeast Extract–Peptone–Dextrose (YPD; Sigma Chemical Corp., St. Louis, MO, USA) liquid medium with AMB (Sigma Chemical Corp., St. Louis, MO, USA) dissolved in 5% dimethyl sulfoxide (DMSO; Samchun, Pyeongtaek, Korea) to 1600 μg/mL. DMSO at 5% was confirmed not to kill the Candida strains. C646 was purchased from MedChem Express and dissolved in 5% DMSO to obtain 20 mM stock solution.

### 2.2 Antifungal susceptibility testing

The Etest (Liofilchem, Roseto, Italy) was used to determine the MICs of *C. krusei* strains *in vitro* on RPMI-1640 medium following the manufacturer’s instructions (24). Briefly, the surface of the agar plate was swabbed with the adjusted *C. krusei* inoculum suspensions (McFarland turbidity of 0.5 at 530 nm). A test strip consisting of a predefined concentration gradient of antifungal agent was placed on the medium surface. After 24 h of incubation, the Etest MICs (μg/mL) were determined directly from the intersection of the growth ellipse and inhibition zone with the tip of the strip. The results of the Etest were verified by M27-A3 microdilution, as proposed by the Clinical and Laboratory Standards Institute (CLSI), with 100 μL diluted suspension (1:50) and 100 μL serial dilutions of each antifungal agent added to each well of 96-well microplates subsequently incubated at 35°C for 24 h.

### 2.3 Induction of the AMB-resistant strain

The AMB-resistant strain was developed by culturing with increasing concentrations of AMB as previously reported (25, 26). Briefly, a single colony of Z748 was transferred to YPD liquid medium without AMB, and incubated at 35°C with shaking for 24–36 h until the medium became turbid. The suspension was centrifuged at 3000 g for 2 min, washed twice, and adjusted to 0.5 McFarland (1–5 × 10^6^ CFU/mL) with normal saline. Subsequently, 200 μL of the solution was added to 20 mL YPD liquid medium containing 0.25 μg/mL AMB for subculture as described above. After 24–48 h of incubation, the cells were subcultured five times in fresh YPD medium containing 0.25 μg/mL AMB. The concentration of AMB was then doubled to 0.5 μg/mL, 1 μg/mL, 2 μg/mL, and so on, till 32 μg/mL. The resistant strain was considered successfully established when it survived 32 μg/mL AMB. The MICs of the AMB-resistant strain were determined by the Etest and ATB Fungus 3, and the resistant strain was subcultured 10 times in YPD liquid medium without AMB to evaluate the stability of the phenotype.

### 2.4 Morphological characteristics and growth curves

Scanning electron microscopy (SEM) was used to evaluate the morphological differences between the AMB-resistant and parent strains. SEM detection was conducted through fixation with 2.5% glutaraldehyde and critical-point drying using CO_2_. The *in vitro* growth kinetics of the Z748R and Z748 strains were evaluated as previously described (27). Briefly, 200 μL of the Z748 or Z748R strain in RPMI-1640 medium (2 × 10^3^ CFU/mL) at pH 7.4 was added to each well of a flat-bottom 96-well microdilution tray. The growth inhibition curve was evaluated by measuring the optical density (OD_600_ nm) every hour over 48 h using a spectrophotometer (Thermo Fisher Scientific, Madrid, Spain). All isolates were tested in triplicate.

### 2.5 Protein extraction

Culture samples were frozen with liquid nitrogen, ground to a powder, and mixed with four times the volume of lysis buffer (1% SDS, 1% protease inhibitor cocktail, 3 μM TSA, and 50 mM NAM), followed by three minutes of ultrasonication on ice using a high-intensity ultrasonic processor (Scientz-IID, Ningbo Xinzhi Biotechnology Co., LTD). To remove the remaining debris, the solution was centrifuged at 12000 g and 4°C for 10 min and the supernatant was transferred to a new centrifuge tube to determine the protein concentration using a BCA kit according to the manufacturer’s instructions.

### 2.6 Trypsin digestion

To precipitate the proteins, samples were slowly treated with tricarboxylic acid (TCA; final concentration of 20% m/v), vortexed, and incubated for 2 h at 4°C. The precipitate was collected after centrifugation at 4500 g and 4°C for 5 min. Protein precipitates were washed three times with pre-cooled acetone and dried for 1 min. Following treatment with 200 mM TEAB, the protein sample was ultrasonically dispersed. For the first digestion, trypsin (1:50 trypsin-to-protein mass ratio) was added and left overnight. After reduction with 5 mM dithiothreitol for 60 min at 37°C, the sample was alkylated with 11 mM iodoacetamide for 45 min at room temperature. In the final step, a Strata X SPE column was used to desalinate the peptides.

### 2.7 Affinity enrichment

Tryptic peptides dissolved in NETN buffer were incubated with pre-washed antibody beads at 4°C overnight with gentle shaking to enrich Ksc modified peptides. The beads were washed with NETN buffer (100 mM NaCl, 1 mM EDTA, 50 mM Tris-HCl, 0.5% NP-40, pH 8.0) four times and H_2_O twice. We used 0.1% trifluoroacetic acid to elute the bound peptides from the beads. The eluted fractions were combined and vacuum-dried before being desalted with C18 ZipTips (Millipore) for liquid chromatography–tandem mass spectrometry (LC–MS/MS) analysis according to the manufacturer’s instructions.

### 2.8 LC–MS/MS analysis

Solvent A (0.1% formic acid and 2% acetonitrile in water) was used to dissolve the tryptic peptides, which were then directly loaded onto a custom reversed-phase analytical column (25 cm length, 100 μm diameter). A gradient of 6% to 22% of solvent B (0.1% formic acid in acetonitrile) was used to separate the peptides over 42 min, 22% to 30% for 12 min, and up to 80% for 3 min, with a final step at 80% for 3 min. All operations were performed at a constant flow rate of 450 nL/min on a nanoElute ultra-high performance liquid chromatography system (Bruker Daltonics). The liquid phase gradient was set as follows: 0 to 43 min, 6%–24% B; 43 to 55 min, 24%–33% B; 55 to 58 min, 33%–80% B; 58 to 60 min, 80% B; and the flow rate was maintained at 450 nL/min.

The peptides were subjected to a capillary source followed by timsTOF Pro (Bruker Daltonics) MS. The electrospray voltage was set to 1.65 kV. The TOF detector was used to analyze precursors and fragments with a MS/MS scan range of 100–1700 m/z. The Bruker timsTOF Pro was operated in parallel accumulation serial fragmentation (PASEF). For fragmentation, precursors with charge states of 0 to 5 were selected and 10 PASEF-MS/MS scans were used per cycle with a dynamic exclusion of 24 s.

### 2.9 Database searches

The MaxQuant search engine (v.1.6.15.0) was used to process the resulting MS/MS data. Tandem mass spectra were searched against the Pichia_kudriavzevii_4909_PR_20210527.fasta database (5,146 entries) concatenated with a reverse decoy database. Trypsin/P was defined as cleavage enzyme allowing up to four missing cleavages and 20 ppm was set as the mass tolerance for precursor ions both in the first and main search. The mass tolerance for fragment ions was set to 20 ppm. Carbamidomethyl on Cys was specified as the fixed modification, and acetylation on N-terminal of protein, oxidation on Met, and succinylation on Lys were specified as variable modifications. False discovery rate was adjusted to < 1%.

### 2.10 Parallel reaction monitoring (PRM) analysis

Peptide samples were prepared as described in Section 2.8. Three independent biological replicates were performed. We used 0.1% formic acid (solvent A) to dissolve the tryptic peptides, which were then loaded onto the custom reversed-phase analytical column. The peptides were separated using a gradient of 6–23% solvent B (0.1% formic acid in 98% acetonitrile) for 38 min, 23–35% for 14 min, and up to 80% for 4 min, with a final step of 80% for 4 min; a constant flow rate was maintained at 700 nL/min using an EASY-nLC 1000 ultra-performance liquid chromatography (UPLC) system.

Peptides were subjected to an NSI ion source for ionization followed by MS/MS in Q Exactive Plus (Thermo Fisher Scientific) coupled to the UPLC. The electrospray voltage was set to 2.0 kV. For a full scan, the m/z scan range was set to 350–1000, and intact peptides were detected in an Orbitrap at a resolution of 35,000. Peptides were selected for MS/MS with the NCE set as 27 and the fragments were detected in the Orbitrap at a resolution of 17,500. We conducted a data-independent procedure alternating between one MS scan followed by 20 MS/MS scans. Automatic gain control was set at 3E6 for full MS and 1E5 for MS/MS. The maximum IT was then set at 20 ms for full MS and auto for MS/MS. We set the isolation window for MS/MS to 2.0 m/z.

We processed the MS data using Skyline (v.3.6). Peptide settings were set as follows: the enzyme was set as Trypsin [KR/P] and Max missed cleavage as 2. The peptide length was set as 8–25, Variable modification was set as Carbamidomethyl on Cys and oxidation on Met, and max variable modifications was set as 3. For transition settings: precursor charges were set as 2, 3; ion charges were set as 1, 2; and ion types were set as b, y, p. The product ions were set from ion 3 to the last ion, the ion match tolerance was set as 0.02 Da.

### 2.11 Functional enrichment analysis

Gene Ontology (GO) annotation was performed using the UniProt-GOA database (http://www.ebi.ac.uk/GOA/) and complemented with InterProScan software (a sequence analysis application). GO protein annotation was based on three categories: biological process, cellular component, and molecular function. Domain Annotation refers to a conserved part of a given protein sequence and structure that can evolve, function, and exist independently of the rest of the protein. InterProScan was used to annotate the protein domain functional descriptions based on protein sequence alignment using the InterPro (http://www.ebi.ac.uk/interpro/) database. The pathways related to the proteins were annotated using the Kyoto Encyclopedia of Genes and Genomes (KEGG) database. We used a two-tailed Fisher’s exact test for each category to determine the level of enrichment for each protein, with significance set at *P* < 0.05. The software motif-X was used to analyze amino acid sequence models surrounding the acetylated lysines.

### 2.12 Western blot (WB)

Proteins were dissolved in a 12% SDS-PAGE gel, transferred to PVDF membranes (Bio-Rad Laboratories, Hercules, CA, USA), and incubated in blocking buffer for 2 h. Subsequently, the membrane was incubated with pan anti-propionyl-lysine, anti-crotonyl-lysine, anti-succinyl-lysine, anti-2-hydroxyisobutyryl-lysine, and anti-β-hydroxybutyryl-lysine antibodies at 4°C overnight. Then, the membrane was incubated with horseradish peroxidase-conjugated goat-antirabbit secondary antibody for another 2 h at room temperature.

### 2.13 Checkerboard microdilution test

The CLSI M27-A3 broth microdilution method was used to determine the MICs of C646 (from 78 μM to 10 mM) and AMB (from 0.125 to 16 μg/mL) against *C. krusei* isolates. We also assessed the fractional inhibitory concentration index (FICI) for C646 plus AMB, which was calculated using a checkerboard microdilution assay. The FICI was interpreted as follows: synergy, < 0.5; additive effect, 0.5–1.0; indifference, 1.0–2.0; antagonism, > 2.0. The experiment was independently repeated three times.

## 3 Results

### 3.1 Generation of AMB-resistant *C. krusei* and its biological characteristics

**Figure 1:**
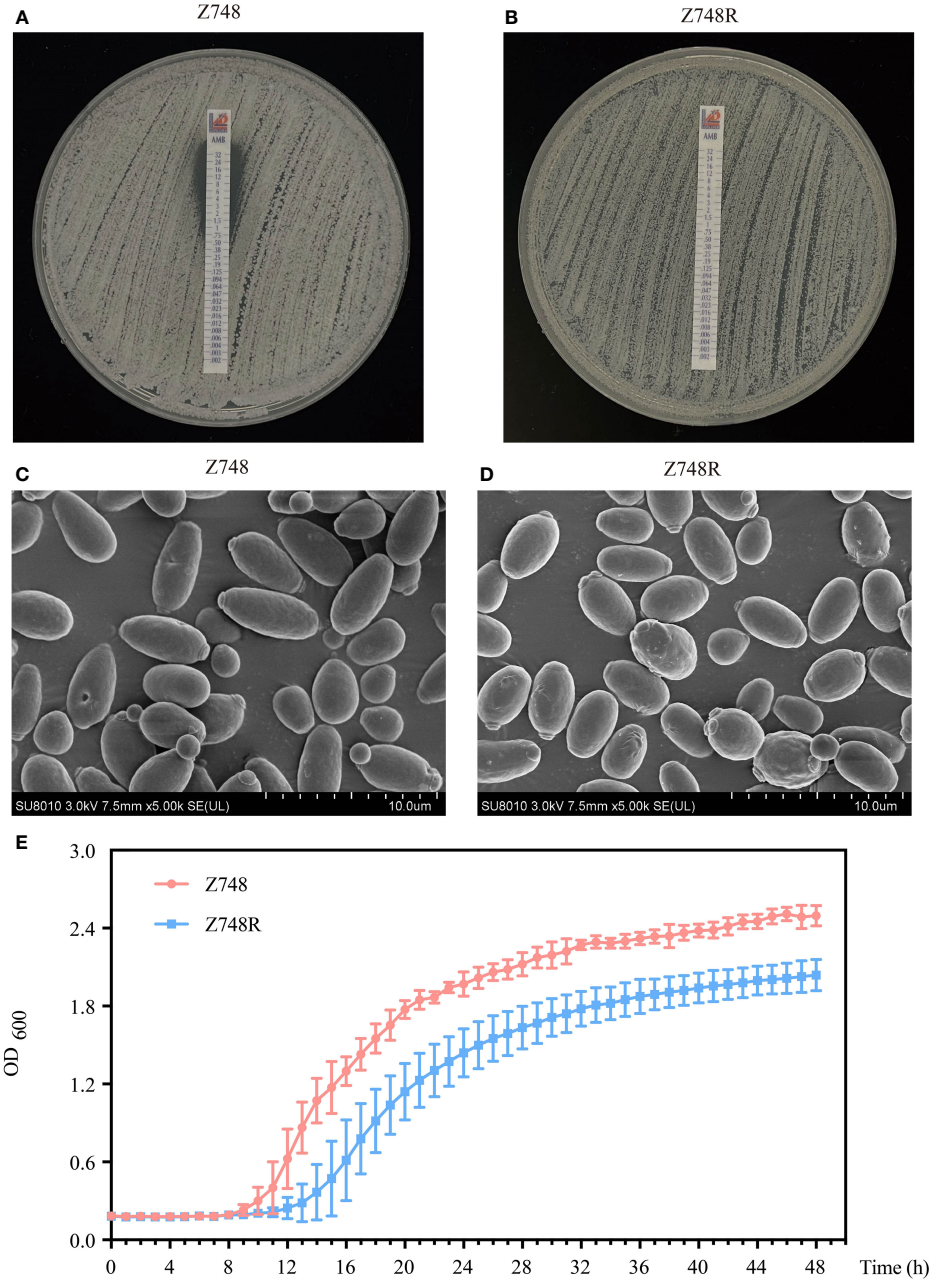
Phenotypic changes between AMB-sensitive *C*. *krusei* (Z748) and the AMB-resistant strain (Z748R). **(A, B)** MIC values of Z748 **(A)**, Z748R **(B)**. **(C, D)** Morphological characteristics of Z748 **(C)** and Z748R **(D)** under a scanning electron microscope. **(E)** 48-h growth curve of Z748 and Z748R.

AMB-sensitive *C. krusei* (Z748) was isolated from the peripheral blood of a 71-year-old male patient with neutropenia at the Shanghai Pulmonary Hospital. The AMB-resistant strain (Z748R) was generated by exposing the parental strain to gradually increasing concentrations of AMB (up to 32 µg/mL). The Etest showed that the MIC of AMB against Z748R increased from 1 μg/mL (**Figure 1A**) to 32 μg/mL (**Figure 1B**); the induced AMB tolerance remained stable after 10 successive passages in an AMB-free environment (**Figure S1**). These results were verified using ATB Fungus 3 (France, BioMérieux) strips.

Scanning electron microscopy showed that Z748 cells were oval and regular with intact membranes (**Figure 1C**), while Z748R cells became round and irregular in shape, and several irregular bulges appeared on the surface of some cells (**Figure 1D**). The 48-h growth curve showed that the lag period of Z748R was longer than that of the sensitive strain Z748; Z748R entered the logarithmic growth phase relatively late. At 20–24 h, both strains entered the platform stage, but the growth curves remained parallel, and the maximum OD value of Z748R was significantly lower than that of Z748, suggesting that the physiological and metabolic characteristics of Z748R might have changed with the acquisition of AMB resistance (**Figure 1E**).

Regarding the response to other antifungal agents, we found that Z748R was more sensitive to azoles, including fluconazole (FLU), itraconazole (ITC), posaconazole (POS), and voriconazole (VOR) (**Figure S2**), compared with that of Z748, suggesting that the drug resistance of the Z748R strain is specific to AMB and not due to common drug resistance mechanisms, such as thickened cell walls or increased efflux pump activity.

### 3.2 Lysine succinylation is associated with amphotericin B resistance in *C. krusei*

Considering the biological changes of the Z748R strain, we explored the molecular mechanism of AMB resistance at the level of protein lysine acylation using a broad-spectrum acylation inhibitor, C646. The checkerboard test revealed that C646 and AMB acted synergistically against *C. krusei* strains (**Table 1**). For C646 combined with AMB (**Table 1**), FICI values for all the strains were < 0.5, indicating that C646 could synergize the antifungal activity of AMB in *C. krusei*.

**Table 1:** Effect of C646 on the antifungal activity of AMB in *C. krusei*.

Isolate	MIC^alone^		MIC^combination^	FICI	Outcome
	AMB (μg/mL)	C646 (mM)	AMB/C646		
Z748	0.5	0.625	0.063/0.039	0.19	synergistic
Z571	1	0.625	0.125/039	0.19	synergistic
Z183	2	0.625	0.25/0.078	0.25	synergistic

AMB, amphotericin B; MIC, minimum inhibitory concentration; FICI, Fractional inhibitory concentration index.

**Figure 2:**
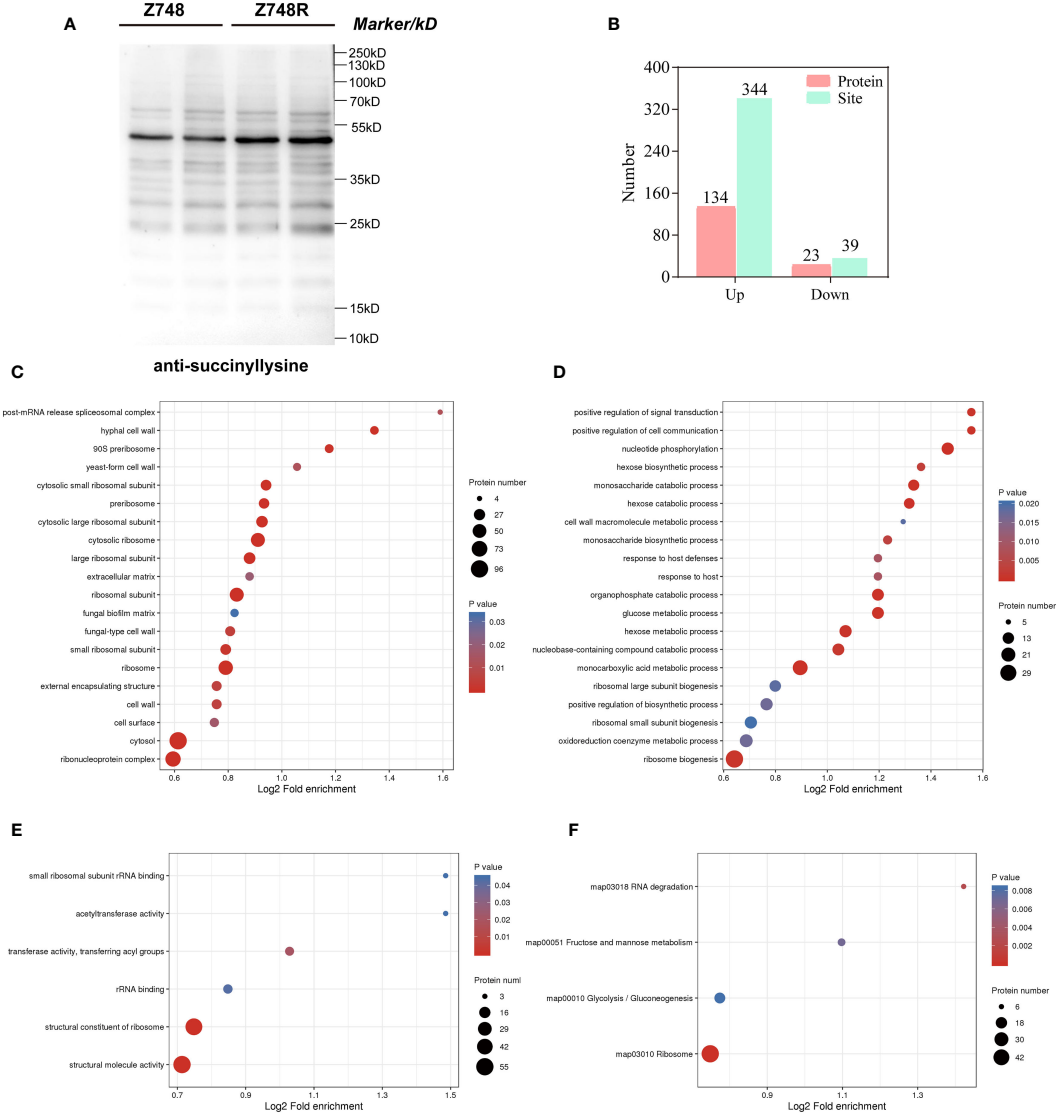
Comparative succinylation analyses between Z748 and Z748R. **(A)** WB analysis showed increased expression of lysine succinylation in Z748R. **(B)** Differentially expressed lysine succinylated proteins and sites. **(C–E)** GO enrichment analysis of differentially expressed succinylated proteins, including cellular components **(C)**, biological processes **(D)**, and molecular functions **(E)**. **(F)** KEGG pathway enrichment analysis of the differentially expressed succinylated proteins.

To determine the difference in lysine acylation between Z748R and Z748, we performed a WB analysis with five broad-spectrum acylation modification antibodies and found that the five novel acylation modifications occurred in both AMB-susceptible and -resistant strains (**Figures 2A**, **S3**). Meanwhile, the overall level of succinylation in Z748R was higher than that in Z748, suggesting that succinylation may be an important driver of AMB resistance in *C. krusei*.

### 3.3 Proteome-wide analysis of lysine succinylation between AMB-resistant and -sensitive strains

We assessed the differentially succinylated proteins (DSPs) and sites between Z748R and Z748 using succinylation modification omics technology. A total of 157 proteins with differentially expressed succinylated sites were identified (*P* < 0.05, fold change [FC] > 1.5 or FC < 0.667), including 344 upregulated sites in 134 proteins, and 39 downregulated sites in 23 proteins (**Figure 2B**). Most differentially expressed succinylated sites were upregulated in Z748R, further suggesting that succinylation may be critical to AMB resistance.

To elucidate the functional implications of DSPs and identify the protein targets for lysine succinylation, we performed GO and KEGG enrichment analyses. GO enrichment of cellular components included the ribosome, ribonucleoprotein complex, cytosolic large ribosomal subunit, and cell wall, suggesting that DSPs were concentrated to the ribosome and cell wall (**Figure 2C**). GO enrichment of biological process included nucleotide phosphorylation, the monosaccharide catabolic process, monocarboxylic acid metabolic process, glucose metabolic process, and positive regulation of cell communication (**Figure 2D**). GO enrichment of molecular functions included structural molecule activity, structural constituent of ribosome, transferase activity, transferring acyl groups, and rRNA binding (**Figure 2E**).

KEGG pathway analysis showed that these proteins were significantly enriched in the ribosome, glycolysis/gluconeogenesis, fructose and mannose metabolism, and RNA degradation (**Figure 2F**). We did not identify any enriched protein domain. These results indicated that a large percentage of DSPs participate in metabolic and translational roles, which could mediate AMB resistance in *C. krusei*.

**Figure 3:**
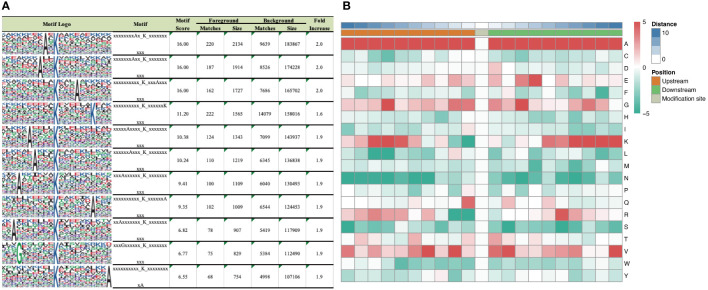
Motif analysis of differentially expressed succinaylated sites. **(A)** Succinaylated sites in particular positions (10 amino acids close to two sides of the succinaylated sites), analyzed using motif-X software. **(B)** Heatmap showing the periodicity of the diverse types of amino acids near the succinylated sites.

According to motif analysis, we found that these differentially succinylated sites matched 11 conserved motifs, namely A*K^Succ^, A**K^Succ^, K^Succ^***A, K^Succ^******K, A****K^Succ^, A***K^Succ^, A******K^Succ^, K^Succ^******A, A*******K^Succ^, G******K^Succ^, and K^Succ^*********A (K^Succ^ represents succinylated lysine residues, * represents any amino acid residues) (**Figure 3A**). By comparing these motifs, we found that four residues—alanine (A), glycine (G), lysine (K), and valine (V)—were ubiquitous around succinylated lysine. When examining the amino acid composition around the succinylated site, we found that there was a higher frequency of alanine at position –10 to +10 (**Figure 3B**).

### 3.4 Proteomic comparison between AMB-resistant and -sensitive strains

**Figure 4:**
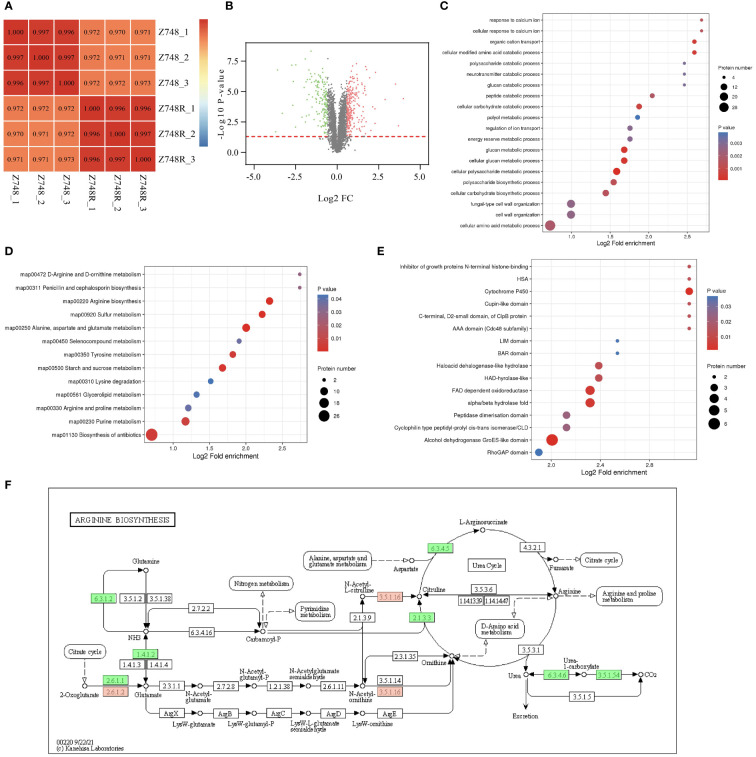
Comparative Proteomic analysis between Z748R and Z748. **(A)** Heatmap of Pearson’s correlation coefficient. **(B)** Volcano plot of DEPs between Z748 and Z748R. **(C)** GO enrichment analysis of DEPs. **(D)** KEGG pathway enrichment analysis of DEPs. **(E)** Protein domain analysis of DEPs. **(F)** DEPs involved in arginine biosynthesis pathway.

Protein PTM can only explain drug resistance to some extent. We performed comparative proteomic analyses between the Z748R and Z748 strains to identify differentially expressed proteins (DEPs) potentially facilitating AMB-related resistance. Pearson correlation analysis of protein expression (**Figure 4A**) showed that the difference of parallel samples within the Z748R and Z748 groups was smaller than that between groups, indicating that the AMB-resistant and the wild-type strain were indeed different in their protein profiles. We identified a total of 415 DEPs (*P* < 0.05, FC > 1.5 or FC < 0.667), among which 252 were upregulated and 163 were downregulated between Z748R and Z748 (**Figure 4B**).

We performed GO functional classification, KEGG pathway, and protein domain enrichment analyses. GO enrichment of cellular components included anchored component of membrane, anchored component of plasma membrane, fungal-type cell wall, and external encapsulating structure, suggesting an association between AMB resistance and aberrant expression of proteins in the cell membrane and wall. GO enrichment of molecular functions included omega peptidase activity, ammonium transmembrane transporter activity, carbohydrate transmembrane transporter activity, and polyamine transmembrane transporter activity. The enriched biological processes included cellular polysaccharide metabolic process, cellular glucan metabolic process, glucan metabolic process, and cellular modified amino acid catabolic process (**Figure 4C**).

KEGG pathway analysis revealed associations between AMB resistance and arginine biosynthesis; alanine, aspartate, and glutamate metabolism; sulfur metabolism; and starch and sucrose metabolism (**Figure 4D**), suggesting a close relationship between AMB resistance and metabolism, particularly amino acid and glucose metabolism. From these results, we inferred that DEPs responsible for metabolism were significantly downregulated (**Figure 4E**). Protein domain analysis of the DEPs showed a significant correlation between AMB resistance and aberrant expression of proteins containing the cytochrome P450 domain (**Figure 4F**).

### 3.5 PRM-based validation

We performed the PRM-targeted protein assay to evaluate the accuracy of the whole-cell proteome and lysine succinylome analyses. A total of 19 proteins (15 upregulated and 4 downregulated) were identified. The qualitative results of the PRM and 4D label-free analyses were highly consistent, especially for trends in protein expression and significant differences (**Tables 2**, **S1**), indicating highly reliable proteomics results. We found that proteins involved in the ergosterol synthesis pathway (A0A099P3X9 and A9YUC7) were significantly upregulated in Z748R. Proteins associated with lysine acylation, especially the writer-histone acetyltransferase (A0A2U9RBF9), were significantly upregulated in Z748R, suggesting that lysine acylation may play a vital role in AMB resistance.

**Table 2:** Comparison of qualitative results between label-free proteomics and PRM.

Accession	Description	FC in label-free	*P*-value	FC in PRM	*P*-value
A0A099P3X9	Cytochrome P450 61	2.2387	5.10^–8^	1.12	4.76^–2^
A9YUC7	Cytochrome P450 lanosterol 14-α-demethylase	1.6704	1.94^–5^	1.14	4.00^–4^
A0A2U9RBF9	Histone acetyltransferase type B catalytic subunit	1.4497	2.76^–3^	1.51	5.89^–5^
A0A099P8F9	Histone deacetylase	1.2207	7.85^–4^	1.16	2.08^–3^
A0A2U9RB90	Glycerol-3-phosphate dehydrogenase [NAD(+)]	1.7375	6.35^–7^	1.72	2.66^–5^
A0A2U9R1G7	Adenylosuccinate synthetase	0.614	2.39^–6^	0.77	4.53^–6^
A0A1V2LUP3	NAD-specific glutamate dehydrogenase	0.4382	3.60^–6^	0.72	1.27^–4^

FC, fold change; PRM, Parallel Reaction Monitoring.

## 4 Discussion

Immunodeficiencies are the main risk factors of fungal infections, which may progress to life-threatening diseases because of difficulties in early diagnosis, lack of appropriate antifungal treatments, and acquired drug resistance (28). *Candida krusei* fungemia occurs most commonly in neutropenic patients with hematologic malignancy and shows poor outcomes despite appropriate antifungal therapy. It is inherently resistant to fluconazole and rapidly develops acquired resistance to other antifungal agents, such as AMB (7). In the present study, we experimentally induced AMB-resistant strains *in vitro* through treatment with escalating AMB concentrations (29–31). We found that the cell morphology, structure, and physiological activities of the AMB-resistant strain changed significantly under the selective pressure of AMB; this phenotype was maintained regardless of the presence of AMB.

Several recent studies have shown that the acylation of key proteins is crucial in the growth, virulence, biofilm formation, and stress resistance of fungi (32–36); moreover, acylation modifications are closely associated with resistance to antibiotics and the immune response (37, 38). Based on the synergistic effect of C646 and AMB against the *C. krusei* strains, we analyzed the differences in acylation modifications between AMB-resistant and -sensitive strains by WB and assessed the importance of succinylation modification in the development of resistance.

Using affinity purification combined with LC–MS/MS, we identified 383 differentially expressed succinylated sites in histones and non-histones, including 344 upregulated sites and 39 downregulated sites, indicating that succinylation levels were enhanced in the AMB-resistant strain compared to those in the parent strain. Four lysine succinylated sites were identified on histone H4, namely K32, K60, K78 and K92; comparative lysine succinylome analysis revealed that the level of K78succ was significantly increased in the AMB-resistant strain compared to that in the parent strain. Previous studies revealed that lysine succinylation can regulate histone function and affect chromatin structure and gene expression (39). Therefore, we inferred that the enhancement of H4K78succ may be related to the mechanism of AMB resistance, though further studies are needed to validate this association.

Lysine succinylation is a conserved PTM that links metabolism and cellular signaling (40). Zheng et al. first described the lysine succinylation map of *C. albicans* in 2016, which showed that lysine succinylation was conserved in many species and played an indispensable role in the regulation of the TCA cycle (41). Increasing evidence indicates that lysine succinylation is central to the metabolism (42), aflatoxin biosynthesis (43), and pathogenicity (44) of fungi. In this study, DSPs (especially those with FC > 2.0) were mainly involved in glycolysis/gluconeogenesis, antibiotic biosynthesis, ketone body synthesis and degradation, carbon metabolism, fructose and mannose metabolism, and amino acid biosynthesis. Our findings suggest that the succinylation of key proteins in the metabolic pathway is associated with the development of AMB resistance in *C. krusei*, which could be useful in the development of targeted strategies to impede AMB resistance.

Given that histone acetyltransferase inhibitors could not fully establish resistance to AMB in *C. krusei*, we considered other potential mechanisms mediating AMB resistance. Using comparative proteome studies, we found that the expression of proteins involved in metabolic processes, especially glucose and amino acid metabolism, changed significantly in the AMB-resistant strain compared to that in the parent strain. Interfering with microbial metabolic homeostasis is an important goal in antibiotic therapy (45, 46). Notario et al. reported a close association between glucan metabolism and AMB-resistance, suggesting that factors promoting glucanase activity could reduce the resistance to AMB, and vice versa (47). In addition, there is a growing body of evidence suggesting that modification of glucose or amino acid metabolic pathways and intermediary metabolic products could rescue microbial drug resistance (48–52). Our study demonstrated that the majority of DEPs involved in glucose or amino acid metabolism were significantly downregulated. Therefore, we inferred that the AMB-resistant strain can resist lethal concentrations of AMB by limiting cell growth, glucose metabolism, and amino acid biosynthesis.

Interestingly, we found that the susceptibility of the AMB-resistant strain to azoles was significantly higher than that of the parent strain, with the greatest sensitivity observed for fluconazole and voriconazole. Although the specific mechanism requires further investigation, these results provide a potential approach for the treatment of *C. krusei* infection in immunodeficient patients following unsuccessful long-term AMB interventions. Future studies are warranted to focus on validating the contributions of differentially succinylated sites in histones and non-histones to drug resistance.

## 5 Conclusion

Our study provides new insights of AMB resistance in *C. krusei*. In particular, we found that enhanced succinylation modification played a vital role in the development of AMB resistance. Proteins related to the metabolic pathway showed a strong association with AMB resistance and could be potential targets for the development of more effective antifungal agents. Our study advances the current paradigm of AMB resistance in *Candida* and provides a basis for the development of antifungal agents for patients suffering from neutropenia and presenting with a weak response to AMB treatment.

## Data availability statement

The data relating to this study was deposited in the iProX repository under accession number: PXD039973. The data can be accessed here: https://www.iprox.cn/page/project.html?id=IPX0005871000.

## Author contributions

WL, WY, and XX designed the study. LZ, JX, and MD collected and analyzed the data. LZ and JX wrote the paper. WL, WY, and XX revised the paper. All authors contributed to the article and approved the submitted version.
