# Supplementary material for: Post-translational modifications confer amphotericin B resistance in Candida krusei isolated from a neutropenic patient
Source: Front Immunol. 2023 Mar 1;14:1148681. doi: 10.3389/fimmu.2023.1148681 (PMC10015421; doi:10.3389/fimmu.2023.1148681)
Supplement: Supplementary file 1 [file DataSheet_1.docx]

Supplementary Material

Post-translational modifications confer amphotericin B resistance in *Candida krusei* isolated from a neutropenic patient

Li Zhang^1†^, Jinzhou Xiao^1†^, Mingwei Du^2,6†^, Wenzhi Lei^1*^, Weiwei Yang^3,4*^, Xiaochun Xue^5*^

^1^ Institute of Dermatology, Naval Medical University, Shanghai, China.

^2^ Department of Cardiology, Shuguang Hospital Affiliated to Shanghai University of Traditional Chinese Medicine, Shanghai, China

^3^Department of Thoracic Surgery, Shanghai Pulmonary Hospital, School of Medicine, Tongji University, Shanghai, China.

^4^Shanghai Engineering Research Center of Lung Transplantation, Shanghai, China.

^5^ Department of Pharmacy, 905th Hospital of PLA Navy, Shanghai, China.

^6^Shanghai Key Laboratory of Traditional Chinese Clinical Medicine, Shanghai, China

^†^Li Zhang, Jinzhou Xiao and Mingwei Du have contributed equally to this work and share first authorship.

*Corresponding author:

E-mail: xxc2021@126.com (Xiaochun Xue), E-mail: CaylaYang@163.com (Weiwei Yang), E-mail: leiwenzhi08@126.com (Wenzhi Lei)

# Supplementary Figures and Tables

## Supplementary Figures


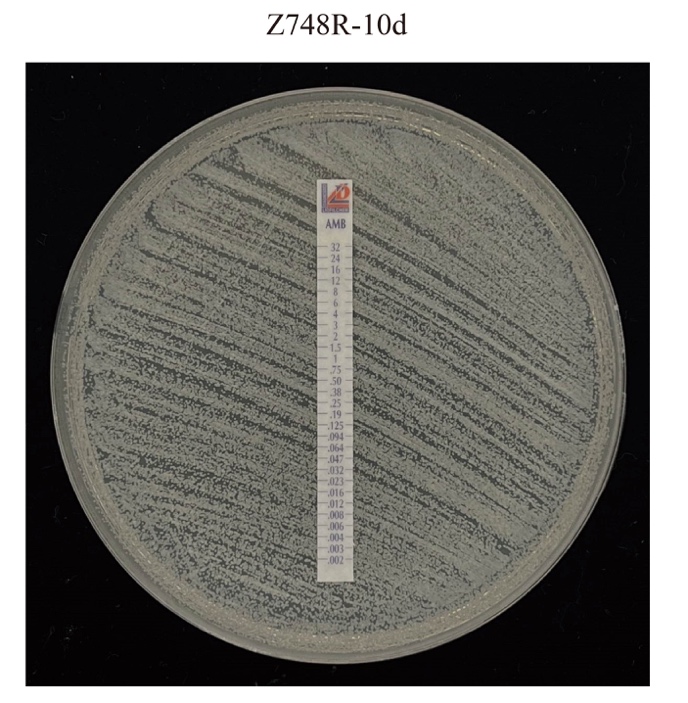


**Figure S1.** Minimum inhibitory concentrations (MICs) of Z748-10d.


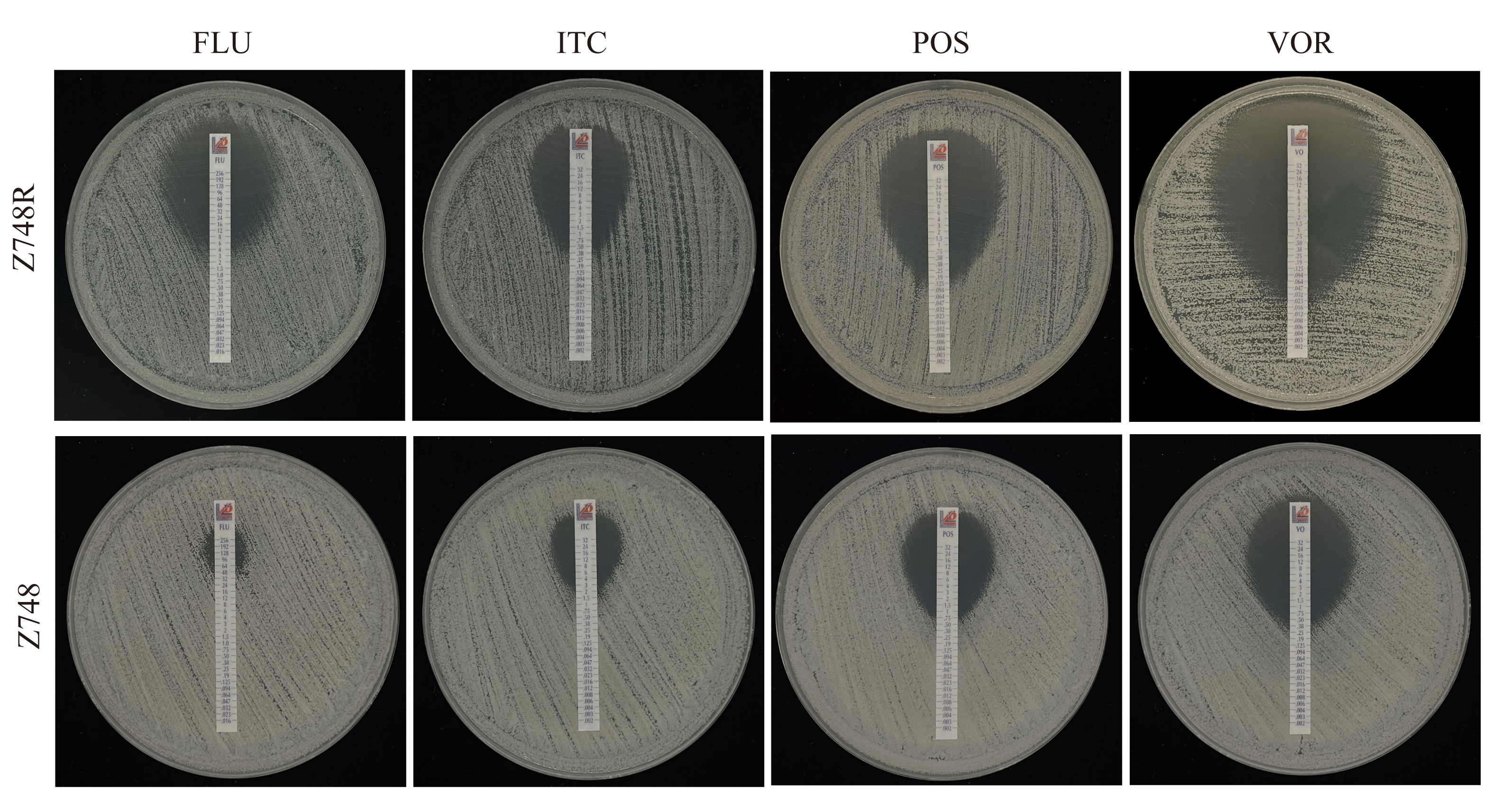


**Figure S2.** Minimum inhibitory concentrations of fluconazole (FLU), itraconazole (ITC), posaconazole (POS), and voriconazole (VOR) against Z748R vs. Z748 (MIC for FLU: 3 μg/mL vs. 48 μg/mL, MIC for ITC: 0.38 μg/mL vs. 2 μg/mL, MIC for POS: 0.125 μg/mL vs. 0.75 μg/mL, and MIC for VOR: 0.016 μg/mL vs. 0.25 μg/mL).


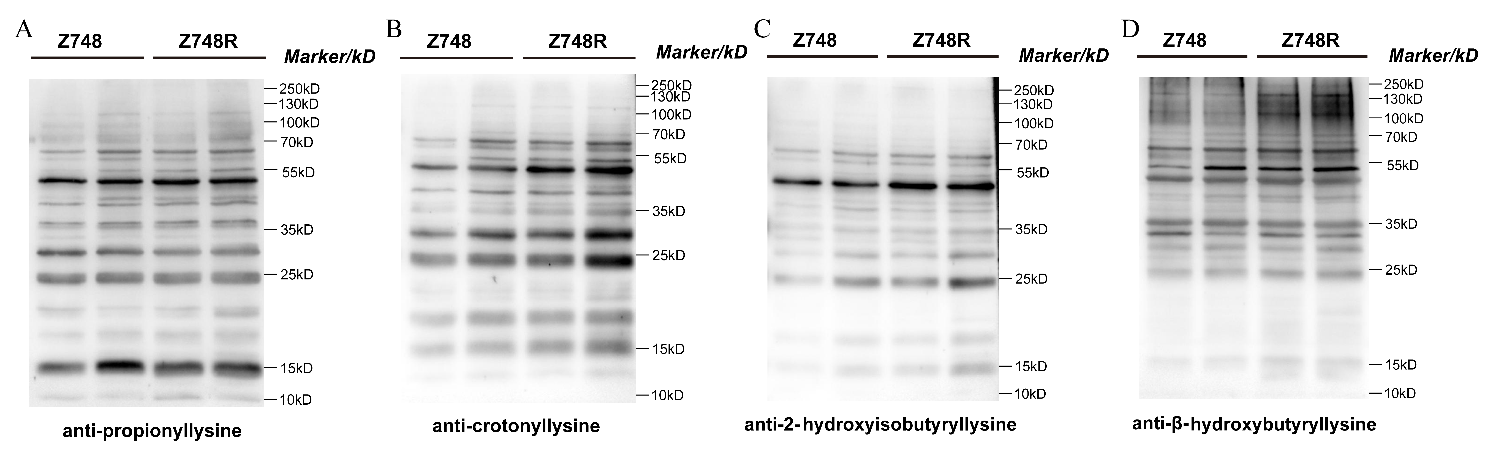


**Figure S3.** Western blot analysis using four pan lysine acylation antibodies, including anti-propionyllysine, anti-crotonyllysine, anti-2-hydroxyisobutyryllysine, and anti-β-hydroxybutyryllysine antibodies.

## Supplementary Tables

Table S1. Comparison of qualitative results between label-free proteomics and parallel reaction monitoring (PRM) analyses.

| Accession | Description | FC in label free | *P* value | FC in PRM | *P* value |
| --- | --- | --- | --- | --- | --- |
| A0A1V2LW54 | Peroxidase | 1.9382 | 1.46^–5^ | 2.37 | 3.01^–6^ |
| A0A099NX85 | Alkaline phosphatase | 1.8428 | 9.43^–4^ | 1.42 | 2.54^–5^ |
| A0A099P4K3 | Calcium/calmodulin-dependent protein kinase | 1.8923 | 7.63^–6^ | 2.5 | 6.90^–8^ |
| A0A2U9R4T3 | Calcium-transporting ATPase | 1.5903 | 1.16^–4^ | 1.66 | 9.04^–6^ |
| A0A1V2LHY8 | GTP:AMP phosphotransferase, mitochondrial | 1.801 | 3.37^–5^ | 1.39 | 1.60^–4^ |
| A0A1V2LN92 | Putative aarF domain-containing protein kinase 1 | 1.5497 | 7.78^–4^ | 1.51 | 1.48^–5^ |
| A0A1V2LUE6 | Aspartic proteinase 3 | 1.7085 | 4.81^–6^ | 2.23 | 9.50^–5^ |
| A0A1Z8JNH7 | CRAL-TRIO domain-containing protein | 1.7657 | 2.74^–4^ | 2.49 | 4.13^–6^ |
| A0A2U9R0D9 | Inositol-3-phosphate synthase | 1.6881 | 2.03^–5^ | 1.52 | 1.26^–3^ |
| A0A2U9R2B5 | E3 ubiquitin protein ligase | 1.7479 | 3.86^–6^ | 1.38 | 1.41^–4^ |
| A0A2U9R2R6 | Uncharacterized protein | 0.5445 | 1.08^–6^ | 0.52 | 1.79^–5^ |
| A0A099P3M1 | Glutamine synthetase | 0.612 | 2.38^–6^ | 0.71 | 6.06^–5^ |
